# Effectiveness of a supervised group exercise therapy based on the biopsychosocial model introduced simultaneously with anti-TNF therapy in anti-TNF-naive patients with active ankylosing spondylitis

**DOI:** 10.55730/1300-0144.5359

**Published:** 2022-01-13

**Authors:** Nur Banu KARACA, Edibe ÜNAL, Jale KARAKAYA, Umut KALYONCU, Sedat KİRAZ

**Affiliations:** 1 Department of Physiotherapy and Rehabilitation, Faculty of Physical Therapy and Rehabilitation, Hacettepe University, Ankara, Turkey; 2 Department of Biostatistics, Faculty of Medicine, Hacettepe University, Ankara, Turkey; 3 Department of Internal Medicine, Faculty of Medicine, Hacettepe University, Ankara, Turkey

**Keywords:** Ankylosing spondylitis, biologic therapy, exercise, psychosocial aspects, TNF-alpha

## Abstract

**Background/aim:**

This study aimed to investigate the effectiveness of a supervised group exercise therapy based on the biopsychosocial model introduced simultaneously with antitumor necrosis factor (TNF) therapy in anti-TNF-naive patients with active ankylosing spondylitis (AS).

**Materials and methods:**

Forty-eight patients were divided into two groups: the control group (CG; n = 36) received only anti-TNF therapy, and the study group (SG; n = 12) received the supervised exercise therapy based on the biopsychosocial model in addition to anti-TNF therapy. The measurements of disease activity and functionality were evaluated by The Bath AS Disease Activity Index (BASDAI) and The Bath AS Functional Index (BASFI) respectively. Other outcome measures evaluated biopsychosocial status, emotional state, spinal mobility, pain, fatigue, sleep, and quality of life. All measurements were applied to both groups at baseline and repeated 12 weeks later.

**Results:**

BASDAI and BASFI analyses revealed significant differences between groups in favor of the SG (p < 0.05). At the end of the 12 weeks, the results showed that there were additional improvements in all outcome measurement parameters in the SG compared to the CG.

**Conclusion:**

The supervised group exercise therapy based on the biopsychosocial model introduced simultaneously with anti-TNF therapy is more effective than only anti-TNF therapy in anti-TNF-naive patients with active AS.

## 1. Introduction

Ankylosing spondylitis (AS) is a chronic, inflammatory, common rheumatic disease of unknown etiology, mainly affecting the sacroiliac joints and the axial skeleton. It can also affect peripheral joints, tendons, ligaments, and extraarticular organs [1,2]. The course of the disease may vary as symptoms are observed in a wide range, from mild to severe [3]. The main complaints of the patients with AS are pain, joint stiffness, fatigue, and functional limitations, which can significantly affect the quality of life and job productivity [4,5]. Patients with AS often experience fatigue, sleep problems, depression, and anxiety, which are the main symptoms that negatively affect daily life [6–9]. Moreover, AS is compatible with the central sensitization mechanism as the nature of chronic pain is an aspect of the biopsychosocial model [10]. All these conditions reveal the importance of biopsychosocial approaches in the treatment of patients with AS [11].

The Assessment in SpondyloArthritis international Society (ASAS) and the European League Against Rheumatism (EULAR) have reported that optimal management in AS is provided by a combination of pharmacological and nonpharmacological treatments. Regular exercise and patient education play a key role in the nonpharmacological treatment of patients with AS [12,13]. Although the positive effects of exercise have been shown by comprehensive studies, the superiority of different types of exercise regimens is still unclear [13,14]. However, the EULAR recommended that pain should be considered from a biopsychosocial perspective, and its management should be planned in accordance with a biopsychosocial approach [15].

Antitumor necrosis factor-alpha (TNFα) therapy, which is often used in patients who do not benefit from routine pharmacological treatment, creates a remarkable difference especially in the treatment of axial involvement, peripheral arthritis, and enthesitis. Some studies have also shown positive effects on quality of life, spinal mobility, depression, and C-Reactive Protein levels [9, 12,16]. Due to the extraordinary advances, this therapy has created in AS management, investigation of the additional beneficial effects of exercise programs alongside anti-TNF therapy has become significant [17]. In this direction, some studies have shown these additional positive effects. However, in almost all of these studies, patients who participated in exercise therapy were anti-TNF-experienced [18]. There is no study in the literature investigating these effects based on the biopsychosocial model in patients with active AS who have not used anti-TNF agents before. Therefore, our study aimed to investigate the effectiveness of a supervised group exercise therapy based on the biopsychosocial model introduced simultaneously with anti-TNF therapy, primarily on disease activity and functionality, in anti-TNF-naive patients with active AS.

## 2. Materials and Methods

### 2.1. Subjects

Seventy-eight patients diagnosed with AS according to the Modified New York Criteria and aged 18–65 years were registered in the study. Subjects were eligible to participate in the study if they were anti-TNF-naive patients with active AS and they were going to begin this treatment. Exclusion criteria included the presence of severe systemic diseases, pregnancy, exercising regularly, participation in rehabilitation treatment in the previous three months, having mental disorders, another chronic inflammatory disease history, and variations in their biological therapy regimens during the study.

Our center provides service to patients with rheumatic diseases from all over Turkey. Randomization could not be performed entirely in this study, as patients coming from outside the city would not be able to participate in exercise sessions. In this way, the patients were divided into two groups according to whether they lived in the city where the study was performed. Thus, out-of-city patients (n = 62) were invited to the control group (CG), and patients living in the same city as our center (n = 16) were invited to the study group (SG). The study was approved by the Ethical Committee of Hacettepe University (KA-17022) and conducted following the principles of the Declaration of Helsinki. Written consent was obtained from all the participants.

#### 2.1.1. Study group (SG)

Anti-TNF-naive patients in the SG were included in the supervised group exercise therapy based on the biopsychosocial model (named as “Bilişsel Egzersiz Terapi Yaklaşımı” (BETY) in original; “the Cognitive Exercise Therapy Approach” in English). The BETY has been used in patients with AS [19] and it is applied in six stages: (1) meeting with the patient; (2) problem analysis; (3) pain management strategies; (4) change and recovery agreement; (5) core stabilization training; and (6) dance therapy-authentic movement (Appendix A). The patients in the SG participated in group exercise sessions for 12 weeks (3 times a week) following the initiation of the anti-TNF therapy. The weekly group exercise sessions were supervised by a physical therapist and each session took 1 h (Appendix B).

#### 2.1.2. Control group (CG)

The control group receiving only the anti-TNF therapy was reevaluated at the end of 12 weeks.

### 2.2. Measurements

Sociodemographic characteristics including age, sex, body mass index, disease features (duration of symptoms, time since diagnosis), and smoking status were recorded at baseline in both groups. In addition, other measurements were administered in both groups at baseline and 12 weeks later. The measurements were as follows:

**A. Bath AS Functional Index (BASFI):** The BASFI was used to determine the functional levels of the patients. This index has 10 questions evaluating the daily living activities. The total BASFI score ranges from 0 to 10 (higher scores reflect a greater impairment) [20].

**B. Bath AS Disease Activity Index (BASDAI):** The BASDAI was used to determine the disease activity levels of the patients. This index consists of 6 items related to 5 symptoms that occurred during the previous week: fatigue, spinal pain, joint pain/swelling, tenderness, and morning stiffness. The total BASDAI score ranges from 0 to 10 (higher scores reflect greater disease activity). A total score of ≥ 4 indicates that the disease is active [21].

**C. Health Assessment Questionnaire (HAQ):** The HAQ was used to assess the health and disability status. This questionnaire consists of 20 items, and the total score is between 0 and 3 (higher scores reflect a higher disability) [22].

**D. BETY-Biopsychosocial Questionnaire (BETY-BQ):** The BETY-BQ was used to assess the biopsychosocial status. It was developed via feedback received from rheumatic patients who received therapy involving a biopsychosocial model (named the BETY) [23]. The validity, reliability, and responsiveness study of the BETY-BQ was conducted in AS patients. This questionnaire consists of 30 items, and the total score is between 0 and 120. A higher score refers to a poor biopsychosocial status [24].

**E. Hospital Anxiety and Depression Scale (HADS):** The HADS was used to determine the depression and anxiety levels of the patients [25]. This scale consists of 14 items. Odd-numbered items evaluate anxiety, and even-numbered items evaluate depression. As a result, two separate total scores between 0 and 21 are obtained for anxiety and depression. A higher score refers to poor depression and anxiety levels. The cut-off values of the scale were determined as 10 for anxiety and 7 for depression.

**F. Tragus-to-wall distance, lateral lumbar flexion, and cervical rotation:** These three measurements were performed bilaterally to evaluate spinal mobility, and the mean of both sides were recorded. Tragus-to-wall distance and lateral lumbar flexion were measured with a tape measure, and the value was recorded in centimeters (cm). The cervical rotation was measured by a universal goniometer, and the value was recorded in degrees.

**G. Visual Analogue Scale (VAS):** Patients were asked to indicate the level of pain on a 10-cm VAS at rest and in activity. In addition, fatigue levels were measured in the same way (A score of 0 indicates no pain/fatigue and a score of 10 indicates very severe pain/fatigue).

**H. Pittsburgh Sleep Quality Index (PSQI):** The PSQI was used to assess subjective sleep quality [26]. This index consists of 24 items and 7 subscales and the total score is between 0 and 21. A high score is related to poor sleep quality. If the total score is 5 or below, sleep quality is interpreted as ‘good’ and if it is above 5, sleep quality is interpreted as ‘bad’.

**I. Short Form-36 (SF-36):** The SF-36 was used to assess the quality of life [27]. It consists of eight subdimensions and each subdimension is scored separately between 0 and 100. Higher scores indicate better health.

### 2.3. Statistical analysis

Data analyses were performed using the IBM SPSS Statistics 23.0 software package. The normality for continuous variables was checked using the Kolmogorov-Smirnov/Shapiro-Wilk test and histogram graphics. Descriptive statistics are presented as mean values and standard deviation or median and minimum-maximum values for quantitative variables according to normal distribution status, and as number and percentage (%) for qualitative variables. For both groups, the independent-samples t-test was used in parametric data, and the Mann-Whitney U test was used in nonparametric data to determine whether there was a difference between baseline conditions. Categorical variables were analyzed by the Fisher’s exact test. A two-way repeated-measures ANOVA was used to examine the main effects and the interaction between groups and times in case of normal distribution. The Bonferroni test was selected for multiple comparisons. Besides, the independent-samples t-test was used to analyze the difference in the change of parametric data between the two groups after the intervention. The Mann-Whitney U test was used to analyze differences between groups, and the Wilcoxon test was used to analyze differences within groups in case of nonnormal distribution. A p < 0.05 was accepted as statistically significant.

## 3. Results

### 3.1. Initial evaluation

After some of the patients withdrew from the study for various reasons, a total of 48 patients completed the 12-week therapy (Figure). The initial values of the main demographic characteristics and all evaluations of the patients in the SG and CG were similar (Tables 1, 2, and 3). All patients were initially in the active disease period (BASDAI ≥ 4). Considering the cut-off values of the HADS and PSQI, patients in both groups initially showed anxiety and depression and were observed to have low sleep quality.

### 3.2. Effects of intervention

Improvements in BASFI, BASDAI, HAQ, BETY-BQ, and HADS scores were observed in both groups (p^1^). The decrease in these scores over time was significant (p^2^) in both groups; however, there was a difference between groups in terms of the amount of this decrease (p^3^). This difference was stemmed from group-time interaction. While there were no differences between the groups in the initial evaluations of BASFI, BASDAI, HAQ, BETY-BQ, and HADS measurements, there were significant differences in the postintervention evaluations (p^4^). When the descriptive statistics of these scores were analyzed, it was seen that these differences were in favor of the SG (Table 2).

Table 3 summarizes the comparison of baseline and postintervention scores of tragus-to-wall distance, lateral lumbar flexion, cervical rotation, pain, fatigue, the PSQI, and the SF-36 within the groups. While all of these measurements showed significant improvements in the SG (p < 0.05); the improvements in the tragus-to-wall distance, lateral lumbar flexion, pain at rest level, and both social function and emotional role subdimensions of SF-36 were not significant in the CG (p > 0.05). When the differences of these measurements before and after the intervention were compared between the groups, it was seen that the improvements in the SG were greater than those in the CG except for the social function subdimension score of the SF-36 (Table 4).

## 4. Discussion

Our outcome measurement parameters were related to functionality and daily living activities, disease activity, biopsychosocial status, emotional status, spinal mobility, pain, fatigue, sleep, and quality of life. Although both groups presented positive results compared to baseline measurements, a combination of supervised group exercise therapy based on the biopsychosocial model and anti-TNF therapy demonstrated additional improvements in all parameters compared to the control group. Moreover, these improvements in the SG occurred earlier in the same time frame. At the end of 12 weeks, all parameters improved in the SG, while the CG showed no changes in tragus-to-wall distance, lateral lumbar flexion, pain level at rest, and social function and emotional role limitation, which are subdimensions of quality of life.

The BETY was introduced to the literature for the first time in patients with AS who received routine pharmacological treatment. Kisacik et al. have investigated the antiinflammatory effects of the BETY in patients with AS who had never received anti-TNF therapy. At the end of their three-month follow-up study, they reported that the BETY yielded positive effects on symptoms and antiinflammatory results in patients with AS [19]. The BETY was chosen as the biopsychosocial approach in our study since its synergistic effects with routine treatment have been shown before. Biological agents are highly recommended in patients who do not respond to nonsteroidal antiinflammatory drugs or for whom these drugs are contraindicated [28,29]. The guidelines recommend anti-TNF agents as the first line of biologic agents, mainly because of a more widespread clinical experience and superior investigations data with these agents [29]. Therefore, to increase the homogeneity of the included patients, only patients who received anti-TNF therapy as a biologic agent were selected in our study.

Although there is no consensus on optimal exercise training in patients with AS, regular exercise has been strongly recommended by experts. Given the improvements in AS treatment due to the remarkable effects of anti-TNF agents, it is recommended to investigate the additional beneficial effects of exercise therapy [17]. Many studies in the literature have examined the effectiveness of anti-TNF therapy alone. Recently, the possible benefits of anti-TNF therapy combined with exercises have been investigated. Liang et al. showed that combinations of anti-TNF therapy and exercise in anti-TNF-experienced patients caused a significant decrease in BASDAI scores compared to anti-TNF alone in a systematic review [30]. Similar results were obtained in our study. In the SG, BASDAI scores were declined more than those of the CG. Spadaro et al. included 27 patients stabilized with anti-TNF treatment for at least 12 weeks in their study and demonstrated the effectiveness of home-based occupational therapy, which included a comprehensive patient training program and exercise [31]. In particular, according to the BASDAI, it is noteworthy that the control group, which had no active disease at the beginning, indicated the value of the active disease at the end of 16 weeks. This may suggest that nonpharmacological treatments are needed due to the chronic nature of the disease, even if the anti-TNF agent is used in patients with AS. Also, in their study, the range of motion exercise of the spine was recommended as a home program, but the exercises were not detailed in terms of frequency, intensity, and duration. In our study, supervised group exercises were an important part of the treatment. Some patient-specific exercises were given as a home program, and pain management strategies were encouraged in case pain occurs. Masiero et al. conducted a randomized controlled study including 62 stabilized patients with AS who had been receiving anti-TNF treatment for at least 9 months. The group, in which anti-TNF therapy, educational-behavioral patient training, and exercise therapy were given together, showed greater improvements in evaluation parameters such as functionality, disease activity, and spinal mobility. In this study, it was emphasized that pharmacological treatment alone could not prevent the need for exercise due to the additional improvements obtained with combined therapy [32]. Their study is similar to our study in terms of having results emphasizing the importance of exercise; however, in that study, psychosocial dimensions of an exercise program were not investigated in anti-TNF-naive active AS patients. In our study, anxiety and depression levels, sleep problems, and the quality of life of these patients were also evaluated. In all these measurements, the SG showed greater improvement than the CG. Yiğit et al. reported that the home exercise program was effective on disease activity, functionality, spinal mobility, depression, fatigue, and quality of life in their study, which included 40 stabilized patients with AS who had been receiving anti-TNF treatment for at least 6 months [33]. In other similar studies, the positive effects of a combination of a home exercise program, incentive spirometry exercises, and spa therapy with anti-TNF treatment have been demonstrated [34–36]. All these studies investigating exercise programs in patients with AS receiving anti-TNF therapy included patients who had received anti-TNF treatment for at least three months.

There are very few studies involving simultaneous effects of exercise along with anti-TNF therapy. Coksevim et al. examined the effects of global postural reeducation (GPR) exercises performed simultaneously with anti-TNF therapy in their study involving 60 patients with active AS. After the exercises were taught to the patients by the physiotherapist, the patients were asked to perform these exercises in the form of a home-based program for at least 30 min per session for 5 days a week for three months. The anti-TNF treatment combined with GPR exercises showed greater improvements in pain, function, and mobility [37]. Our study differs from other studies in the literature in terms of using a biopsychosocial approach, which is recommended by the EULAR guidelines [15]. Anti-TNF-naive patients were selected and a physiotherapist-supervised group exercise program was initiated concurrently with medical treatment. Performing the sessions in the form of supervised group exercises may be a factor that increases motivation and socialization. The BETY, which includes pain management, dance therapy-authentic movement, cognitive restructuring about disease, and function-oriented core stabilization exercises, is presented in this study as an effective exercise program when introduced simultaneously with anti-TNF therapy in anti-TNF-naive patients with active AS.

### 4.1. Limitations

There are several limitations of the study such as small sample size and lack of randomization of the participants. In our study, the intermalleolar distance and the lumbar flexion measurements could not be made due to the lack of a suitable environment for the control group’s evaluation. Therefore, the Ankylosing Spondylitis Metrology Index score for mobility assessment could not be obtained. Considering the positive effects of anti-TNF agents at 16–24 weeks on ASAS response, the longer duration of the study might have resulted in more accurate interpretations. Further studies with longer follow-up and larger sample sizes are needed.

### 4.2. Conclusion

It was concluded that the supervised group exercise therapy based on the biopsychosocial model introduced simultaneously with anti-TNF therapy is more effective than only anti-TNF therapy in anti-TNF-naive patients with active AS. In addition, this original study provides an example of a biopsychosocial model suitable for clinical practice in this patient group.

## Figures and Tables

**Figure:**
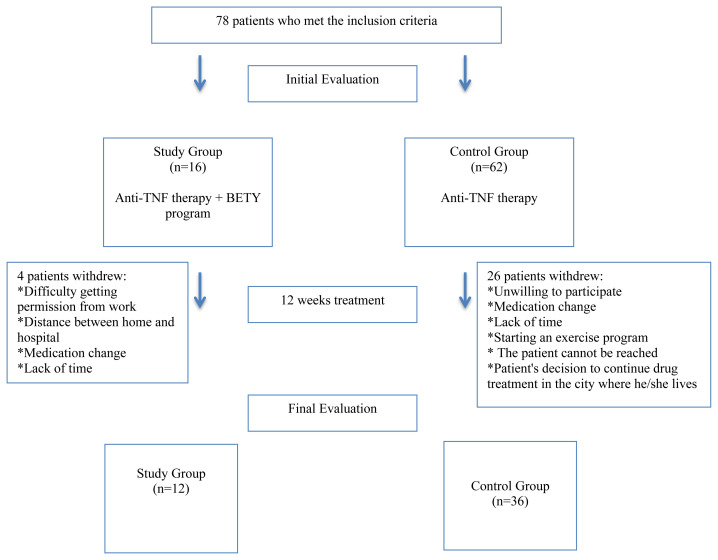
Patients’ flow diagram.

**Table 1:** Main demographic and clinical features of AS patients in the study and control groups.

	SG (n = 12)	CG (n = 36)	Independent samples t-test p
Characteristic	χ̄ ± SD	χ̄ ± SD	
Age (year)	38.75 ± 9.52	39.31 ± 9.89	0.866
Height (cm)	163.17 ± 5.73	163.60 ± 10.33	0.891
Weight (kg)	70.42 ± 12.16	71.4 ± 16.52	0.851
BMI (kg/m^2^)	26.41 ± 4.10	26.51 ± 4.82	0.948
	Median (min-max)	Median (min-max)	Mann-Whitney U test p
Disease duration (years)	2.00 (0–21)	2.00 (0–30)	0.862
Symptom duration (years)	11.00 (2–25)	8.00 (0–35)	0.200
Delay time for diagnosis (years)	5.50 (1–15)	5.00 (0–15)	0.298
Sex	n (%)		Fisher’s Exact Test
Female (%)	10 (83.3)	21 (58.3)	0.169
Male (%)	2 (16.7)	15 (41.7)	
Smoking	n (%)		
Yes (%)	2 (16.7)	14 (38.9)	0.289
No (%)	10 (83.3)	22 (61.1)	

BMI: Body mass index χ̄: Mean, SD: Standard deviation

**Table 2:** Baseline and postintervention scores of the BASFI, BASDAI, HAQ, BETY-BQ, and HADS within and between the groups.

	Initial evaluation	Final evaluation	p^1^	p^2^	p^3^
BASFI (0–10)	χ̄ ± SD	χ̄ SD			
SG	5.68 ± 1.44	1.52 1.08	<0.001	<0.001	0.01
CG	5.13 ± 2.43	3.52 ± 2.19	<0.001		
p^4^	0.464	0.04*			
BASDAI (0–10)	χ̄ ± SD	χ̄ ± SD	p^1^	p^2^	p^3^
SG	6.01 ± 1.56	1.84 ± 1.07	<0.001	<0.001	<0.001
CG	6.12 ± 1.77	4.49 ± 2.13	<0.001		
p^4^	0.851	0.00*			
HAQ (0–3)	χ̄ ± SD	χ̄ ± SD	p^1^	p^2^	p^3^
SG	1.20 ± 0.47	0.29 ± 0.29	<0.001	<0.001	0.01
CG	1.17 ± 0.69	0.80 ± 0.60	<0.001		
p^4^	0.893	0.007*			
BETY-BQ (0–120)	χ̄ ± SD	χ̄ ± SD	p^1^	p^2^	p^3^
SG	66.25 ± 20.57	20.67 ± 10.14	<0.001	<0.001	<0.001
CG	59.33 ± 25.14	44.28 ± 23.23	<0.001		
p^4^	0.394	0.001*			
HADS-A (0–21)	χ̄ ± SD	χ̄ ± SD	p^1^	p^2^	p^3^
SG	12.33 ± 4.73	3.33 ± 1.67	<0.001	<0.001	<0.001
CG	10.33 ± 5.38	7.72 ± 4.70	<0.001		
p^4^	0.258	0.003*			
HADS-D (0–21)	χ̄ ± SD	χ̄ ± SD	p^1^	p^2^	p^3^
SG	9.67 ± 3.62	2.75 ± 2.26	<0.001	<0.001	0.001
CG	8.89 ± 4.70	5.78 ± 4.70	<0.001		
p^4^	0.604	0.038*			

A two-way repeated-measures ANOVA

p^1^: Comparison of the first and second evaluations within groups (pairwise comparison)

p^2^: General comparison of change over time

p^4^: Comparison of first and second evaluations between groups (pairwise comparison)

BASFI: Bath Ankylosing Spondylitis Functional Index; BASDAI: Bath Ankylosing Spondylitis Disease Activity Index; HAQ: Health Assessment Questionnaire; BETY-BQ: BETY-Biopsychosocial Questionnaire; HADS: Hospital Anxiety and Depression Scale; χ̄: Mean; SD: Standard Deviation.

* Significant at p < 0.05.

**Table 3:** Baseline and postintervention scores of tragus-to-wall distance, lateral lumbar flexion, cervical rotation, pain, fatigue, PSQI, and SF-36 within the groups.

		Initial evaluation	Final evaluation	Wilcoxon test
		Median (min-max)	Median (min-max)	p
Tragus-to-wall distance (cm)	SG	17.25 (13.0–23.5)	13.2 (10.0–21.0)	0.002*
	CG	17.0 (10.0–26.5)	16.5 (7.0–26.0)	0.554
p*		0.933		
Lateral lumbar flexion (cm)	SG	8.0 (5.0–15.0)	15.5 (10.0–20.0)	0.002*
	CG	8.75 (2.0–21.0)	9.0 (2.0–21.0)	0.897
p*		0.633		
Cervical rotation (°)	SG	53.75 (6.5–62.5)	63.75 (32.5–85.0)	0.002*
	CG	45.0 (7.5–65.0)	45.0 (15.0–70.0)	<0.001*
p*		0.212		
Pain-at rest (VAS, cm) (0–10)	SG	7.0 (3–9)	3.0 (0–4)	0.002*
	CG	7.0 (2–10)	4.0 (0–9)	0.221
p*		0.818		
Pain-in the activity (VAS, cm) (0–10)	SG	6.0 (4–10)	1.5 (0–4)	0.002*
	CG	7.0 (1–10)	4.0 (0–9)	0.000*
p*		0.916		
Fatigue (VAS, cm) (0–10)	SG	7.5 (5–10)	3.0 (0–4)	0.002*
	CG	8.0 (3–10)	6.0 (1–10)	<0.001*
p*		0.627		
PSQI (0–21)	SG	13.0 (7–17)	1.0 (0–6)	0.002*
	CG	11.0 (0–17)	10.5 (0–16)	0.028*
p*		0.338		
SF-36 (0–100)				
Physical Functioning	SG	38.8 (11.1–83.3)	80.5 (61.1–94.4)	0.002*
	CG	27.7 (0.0–88.8)	66.6 (0.0–100)	<0.001*
p*		0.981		
Role physical	SG	0.0(0–0)	100 (25.0–100.0)	0.002*
	CG	0.0 (0.0–100)	12.5 (0.0–100)	0.004*
p*		0.078		
Bodily pain	SG	45.0 (22.5–67.5)	85.0 (67.5–90.0)	0.002*
	CG	32.5 (10.0–80.0)	66.2 (22.5–100)	<0.001*
p*		0.160		
Social functioning	SG	50.0 (0.0–100)	100 (50.0–100.0)	0.008*
	CG	50.0 (0.0–100)	93.75 (0.0–100)	0.066
p*		0.990		
Mental health	SG	56.0 (24.0–76.0)	80.0 (44.0–88.0)	0.002*
	CG	52.0 (12.0–88.0)	64.0 (24.0–96.0)	<0.001*
p*		0.792		
Role emotional	SG	0.0 (0.0–100)	83.3 (0.0–100)	0.004*
	CG	0.0 (0.0–100)	0.0 (0.0–100)	0.269
p*		0.184		
Vitality	SG	25.0 (0.0–60.0)	60.0 (35.0–80.0)	0.003*
	CG	25.0 (0.0–80.0)	50.0 (10.0–90.0)	<0.001*
p*		0.540		
General health	SG	35.0 (25.0–65.0)	60.0 (35.0–85.0)	0.003*
	CG	40.0 (5.0–90.5)	45.0 (15.0–90.0)	0.034*
p*		0.449		

VAS: Visual Analogue Scale; PSQI: Pittsburgh Sleep Quality Index; SF-36: Short Form-36. p^*^ values for differences in the baseline data between the SG and the CG using the Mann-Whitney-U test.

* Significant at p < 0.05.

**Table 4:** Postintervention difference scores of tragus-to-wall distance, lateral lumbar flexion, cervical rotation, pain, fatigue, PSQI, and SF-36 between the groups.

	SG (n = 12)	CG (n = 36)	p*
	Median (min-max)	Median (min-max)	
Tragus-to-wall distance	3.25 (1.5/6.5)	0.5 (−7/7)	<0.001*
Lateral lumbar flexion	−5.75 (−3/−8)	0.0 (−8.5/8.5)	<0.001*
Cervical rotation (°)	−20.0 (−10/−35)	−2.50 (0/−17.50)	<0.001*
Pain-at rest	4.0 (2/7)	1.0 (−4/10)	0.001*
Pain-in activity	5.0 (3/8)	2.0 (−4/10)	0.001*
Fatigue	5.0 (2/8)	2.0 (−3/6)	<0.001*
PSQI (0–21)	10.5 (6/17)	1.0 (−9/9)	<0.001*
SF-36 (0–100)			
Physical functioning	−38.85 (−11.1/−77.8)	−11.15 (27.7/−66.7)	0.002*
Role physical	−100.0 (−25/−100)	0.0 (75/−100)	0.001*
Bodily pain	−35.0 (−10/−55)	−22.5 (12.5/−87.5)	0.042*
Social functioning	−31.25 (0/−100)	0.0 (−100/100)	0.108
Mental health	−22.0 (−8/−56)	−12.0 (28/−76)	0.050
Role emotional	−66.6 (0/−100)	0.0 (−100/100 )	< 0.001*
Vitality	−32.5 (0/−55)	−22.5 (20/−60)	0.050
General health	−20.0 (0/−55)	−10.0 (35/−40)	0.015*

VAS: Visual Analogue Scale, PSQI: Pittsburgh Sleep Quality Index, SF-36: Short Form-36.

* Significant at p < 0.05. Mann-Whitney-U test

**Appendix A:** Process of the BETY.

**Meeting with the patient:** The characteristics of the disease are explained. The anxiety and fear of the patient regarding their disease are minimized.
**Problem analysis:** The patient is asked about their primary complaint and functional problem regarding their disease. After the problem is identified, exercises that can solve the patient’s complaints are taught. These also form the patient’s home-based exercise program.
**Pain management strategies:** The relationship between pain-muscle spasms and limbic system via central sensitization mechanism; pain coping skills and problem-solving techniques (activity-rest cycling, pleasant activity planning, pleasant imagery, etc.); positive thinking and distraction strategies about pain management are presented.
**Change and recovery agreement:** The patient is asked to restrict the activity in the painful body area and to perform the exercises when pain occurs during the day. Active participation of the patient in the treatment is encouraged and the patient’s awareness about the responsibility towards treatment is increased. Change and recovery agreement is made with the patient who agrees to participate actively.
**Core stabilization training:** Head and neck, shoulders, chest, lumbopelvic region postures and respiratory control, which are features of core stabilization, are explained and taught.
**Dance therapy-authentic movement:** Dance therapy-authentic movement can be applied at the beginning and at the end of the session. Following the commands given by the physiotherapist accompanied by music, the patients are asked for an instant movement called “authentic movement”. At this point, it is aimed to distract the patient’s attention from the pain and the related body area and correct the posture impaired by the muscle spasm.

**Appendix B:** Process the core stabilization exercises.

0–6. weeks			
Warm-up phase	Main exercise training phase (0–3. weeks)	Main exercise training phase (0–6. weeks)	Cool-down phase
•Mini squat	•Hundreds 1–2	·Hundreds 3	·The saw
•Roll down	•One leg stretch	·Shoulder bridge 1	·Mermaid
•PNF patterns-upper extremity	•Double leg stretch	·One leg stretch	·Chest stretch
•Cleopatra	•Shoulder bridge	·Double leg stretch	·Swinging
•Chest stretch	•Swan dive	·Swan dive	
•Swinging	•Swimming	·Swimming	
•Dumb waiter	•Clam	·Clam	
	•Hip twist	·Hip twist	
	•Sidekick	·Sidekick 2	
	•One leg kick 1	·Arm openings 1–2	
	•Roll down		
**6–12. weeks (resistive exercise with exercise band)**			
**Warm-up phase**	**Main exercise training phase (6–9. weeks)**	**Main exercise training phase (9–12. weeks)**	**Cool-down phase**
·Mini squat	·Abdominal preparation	·Hundreds	·Mermaid
·Roll down	·Shoulder bridge 2	·Side circles	·Spine twist
·PNF patterns-upper extremity	·Sidekick press	· Shoulder bridge 2	·Side bend
·Cleopatra	·One leg stretch 2	·One leg stretch 3	·Chest stretch
·Chest stretch	·Parallel (double leg)	·Heels together toes apart	·Crock screw
·Swinging	·Parallel (single leg)	·External rotation	
·Dumb waiter	·Point & Flex	·Diamond press with arm openings	
	·Diamond press	·Roll up with obliques	
	·Breaststroke		

**Note:** The exercises are generally progressed in this way, but differences occurred between patients due to differences in their tolerance levels
